# New insights into the mechanism and DNA-sequence specificity of INO80 chromatin remodeling

**DOI:** 10.21203/rs.3.rs-3443329/v1

**Published:** 2023-10-30

**Authors:** Blaine Bartholomew, Shagun Shukla, Mzwanele Ngubo, Somnath Paul, Jim Persinger, Sandipan Brahma

**Affiliations:** The University of Texas MD Anderson Cancer Center; The University of Texas MD Anderson Cancer Center; Ottawa Hospital Research Institute; UT MD Anderson Cancer Center; The University of Texas MD Anderson Cancer Center; The University of Texas MD Anderson Cancer Center

## Abstract

The INO80 complex stood out in a large family of ATP-dependent chromatin remodelers because of its ATPase domain binding and translocating on DNA at the edge of nucleosomes, rather than at two helical turns from the center of DNA that is wrapped around nucleosomes. This unique property of INO80 was thought to account for its singular role in nucleosome placement at gene promoters in a DNA-sequence dependent manner that is crucial for transcription regulation. Now, we uncover INO80 functions differently than previously thought with its ATPase domain translocating on DNA close to the center of nucleosomes, like other remodelers. Our discovery also reveals the physical properties of the first ~36 bp of DNA on the entry side of nucleosomes is the main determinant for the DNA specificity of INO80 rather than the properties of the extranucleosomal DNA. The DNA sequence sensitive step of INO80 is after DNA is displaced from the histone octamer on the entry side of nucleosomes and 20 bp of DNA are moved out the exit side. We find the ATPase domain and Arp5 subunit of INO80 are likely involved in INO80’s DNA specificity and the mechanism of INO80 remodeling is substantially different than originally proposed.

## Introduction

In 2017, chemical crosslinking and DNA footprinting techniques revealed that the ATPase domain of the INO80 chromatin remodeling complex associates with nucleosomal DNA at the SHL-6 position^1^. Building on this observation, a scan was performed to find where single nucleotide gaps and nicks in nucleosomal DNA interfere with nucleosome movement, given DNA gaps/nicks are known to block DNA translocation of chromatin remodelers^2–5^. Gaps from Superhelical Locations (SHL)-2 to -6, where DNA enters nucleosomes, were found to efficiently block nucleosome mobilization by INO80 and helped confirm the idea that the ATPase domain of INO80 translocates at SHL-6^1^. The following year cryo-EM revealed a mixed population of nucleosome bound *Chaetomium thermophilum* and human INO80 with the ATPase domain bound on nucleosomal DNA at SHL-6 or SHL + 2 positions^6,7^. Given the earlier biochemical data, it was assumed that the structure with the ATPase domain at SHL-6 represented the active form of INO80. Interestingly, the structure of INO80’s ATPase domain bound at SHL-6 differs from that observed with Chd1 and Isw1a ATPase domains bound to SHL + 2 ^7–9^. In the same year, the cryo-EM structure of yeast SWR1 complex-bound nucleosomes revealed its ATPase domain bound to DNA at SHL + 2, quite different than INO80 despite sharing numerous subunits and both belonging to the same sub-family of ATP-dependent chromatin remodelers^10^. Since then, INO80 is the only ATP-dependent chromatin thought to translocate on DNA near the edge of nucleosomes and this assumption has influenced many subsequent studies on the properties of this complex including its DNA sequence specificity.

The DNA sequence specificity of INO80 was found using yeast genomic DNA reconstituted into chromatin and scanning for ATP-dependent chromatin remodeler(s) that could restore the same nucleosome positioning as observed in vivo ^11^. These studies found INO80 alone can correctly position more than 90% of nucleosomes at the + 1-position, next to the transcription start site. INO80 positioning of the + 1 nucleosome is not due to nucleosome spacing, a property that INO80 had been previously shown to exhibit, because the proper positioning of nucleosomes was not dependent on the density of nucleosomes assembled on DNA in these experiments^12,13^. These data show INO80 has intrinsic DNA sequence specificity and the DNA near promoters can arrest INO80 from further mobilizing nucleosomes. Examination of the physical properties of INO80 positioned + 1 nucleosomes compared to salt gradient dialysis (SGD) positioned nucleosome revealed linker DNA had more pronounced DNA propeller twist and the H2A-H2B bound DNA had less twist, corresponding to respectively more and less rigid DNA in the INO80 positioned than SGD nucleosomes^14^. From these experiments, the physical properties of extranucleosomal DNA was thought to restrict INO80 remodeling and is supported by the idea of the ATPase domain translocating on DNA close to where extranucleosomal DNA enters and the Arp8/Arp4/actin module bound to extranucleosomal DNA known to control INO80 nucleosome mobilization activity^15,16^. This premise however was not well experimentally validated when the remodeling activity of nucleosomes with rigid and flexible linker DNA showed only minor differences^17^.

Currently, we find that our view of how INO80’s ATPase domain engages nucleosomes is incomplete and we need to revise the models of INO80 remodeling nucleosomes. Our current data support INO80’s ATPase domain translocating on DNA at SHL + 2 not SHL-6 and the ATPase contacts at SHL-6 being important in regulating INO80’s remodeling activity. The DNA gaps shown earlier now appear to not block translocation but instead likely impact the physical properties of DNA being displaced at this region akin to that seen with DNA sequence specificity^1^. The physical properties of DNA displaced from H2A-H2B on the entry side of nucleosomes are critical for the efficient passage of DNA from the entry to the exit sides of nucleosomes. Although previously overlooked, we find that the DNA sequence bound to H2A-H2B is the primary driver for INO80’s DNA sequence specificity and Arp5’s grappler and DNA “gatekeeping” activity are likely involved.

## Results

### The ATPase domain of Ino80 associates with and translocates on DNA at SHL + 2 rather than at SHL-6

**Figure 1:**
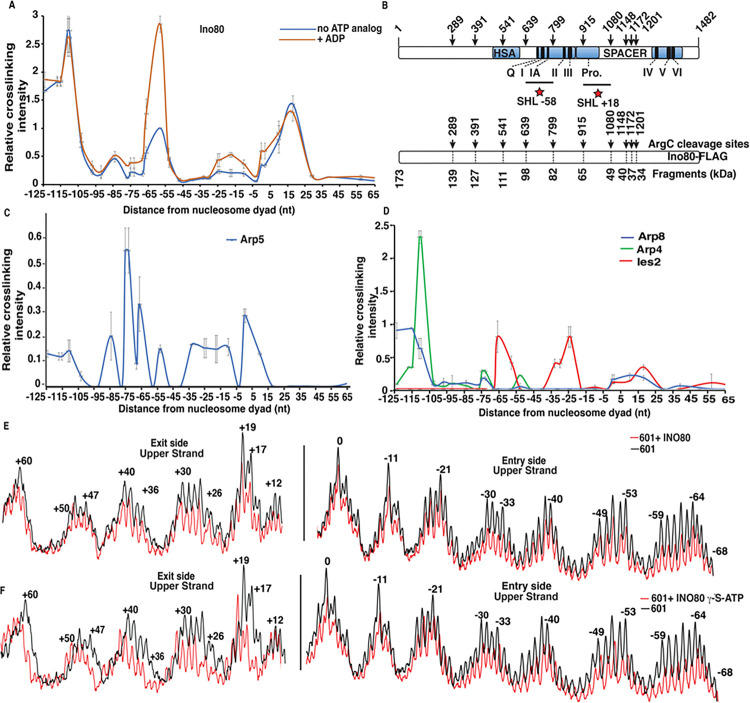
INO80 binds at SHL+2 and SHL-6 and translocates at SHL+2. (A,C,D) Crosslinking efficiencies of INO80 subunits at various nucleotide positions were quantitated from phosphorimages, normalized relative to Ino80 crosslinked at nt-58 and plotted. Values are mean of ≥3 experiments and error bars are ± s.d. (B) The regions of Ino80 crosslinked to DNA at nt-18 and -58 is shown as determined by peptide mapping with Arg-C protease. (E-F) The DNA footprint of INO80 bound to 601 nucleosomes at the exit and entry side is shown with or without the nucleotide analog g-thio-ATP added. Black lines are nucleosome only and red lines are nucleosome with INO80.

INO80 interactions with nucleosomal and extranucleosomal DNA are probed by site-directed DNA photocrosslinking using nucleosomes with 70 and 5 bp of extranucleosomal DNA (70N5) ^1,18^. The side of nucleosomes with the 70 bp extranucleosomal DNA is the (−) side in reference to the dyad axis and the (+) side is on the opposite side of the nucleosomes. In 70N5 nucleosomes the (−) and (+) sides are where DNA respectively enter or exit nucleosomes when remodeled by INO80. Ino80, the catalytic subunit of the INO80 complex, is crosslinked to DNA at a new position from nt + 4 to + 18 from the dyad axis that was not previously observed and at nt −58 and − 110/−111, positions that had been previously reported^16^ (Fig. 1A). Peptide mapping shows the protrusion region proximal to the N-terminal lobe of the ATPase domain is crosslinked to nt + 18 (SHL + 2), a region of the N-lobe distinct from that crosslinked to nt-58 (SHL-6), thus demonstrating the ATPase domain of Ino80 is bound at both SHL + 2 and SHL-6 (Fig. 1B and Supplementary Fig. 1). Arp5 is shown to make extensive contacts with DNA spanning from the dyad axis through the (−) or entry side all the way to the linker or extranucleosomal DNA by DNA crosslinking (Fig. 1C). The DNA crosslinking of Arp5 is consistent with prior cryo-EM data showing the DNA binding domain of Arp5 binding to SHL-2/-3 and the grappler region binding where DNA enters nucleosomes, SHL-1 and the dyad depending on whether it is in the open or closed configuration^6,7,19,20^. Ies2 is also seen to be associated at SHL + 2 along with the ATPase domain consistent with the prior cryo-EM structure^7^ and near SHL-6 and − 2 positions not previously observed. It is not surprising to detect Ies2 interactions not seen in the cryo-EM structure given that the vast majority of Ies2 is not resolved in these experiments.

We model key segments of yeast Ino80 that were absent in the available structures using Alpha Fold2 and align this structure to those of INO80 and SWR1 bound to nucleosomes to find if the region of Ino80 mapped to SHL + 2 and − 6 are consistent with the ATPase domain actively translocating at SHL + 2 ^10,21^. When comparing these models, the site crosslinked at SHL-6, spanning from amino acid 683 to 799, is consistent with both models, making it inconclusive as to which model is more likely correct (Supplemental Fig. 1B and Movie 1). However, the region of Ino80 from amino acid 915 to 1080 is only proximal to the correct site in DNA when the ATPase domain is actively engaged at SHL + 2 and not at SHL-6 (Supplemental Fig. 1B and Movie 2).

DNA footprinting shows INO80 extensively interacting with DNA similar to that observed by DNA crosslinking and highlights where the ATPase domain of Ino80 translocates on nucleosomal DNA. INO80 protects the upper strand of DNA at the SHL-1 to + 3 and SHL - 5/-6 consistent with where the ATPase domain of Ino80 binds (Fig. 1E). There is also strong protection at SHL-2/-3 where the DNA binding domain of Arp5 is known to bind and moderate protection at the dyad axis, SHL-1 and entry site of nucleosome centered at nt-74 corresponding where the grappler domain of Arp5 binds (Figs. 1E and Fig. 4B). DNA footprinting is used to track DNA translocation of INO80 after addition of g-S-ATP, a slow hydrolyzing analog of ATP, by shifting the nucleosomal protection pattern. The nucleosome protection pattern is shifted as expected by 1–2 nucleotides in the vicinity of SHL + 2 on the (+) or exit side of nucleosomes and not on the entry side at SHL-6 (Fig. 1F). These data show the ATPase domain of Ino80 actively translocates on DNA at SHL + 2 and not at SHL-6, consistent with modeling shown above of the regions of the ATPase domain that are crosslinked to both positions.

### Extended DNA movement by INO80 on the exit side of nucleosomes coincides with DNA lifting off from the histone octamer on the entry side.

**Figure 2:**
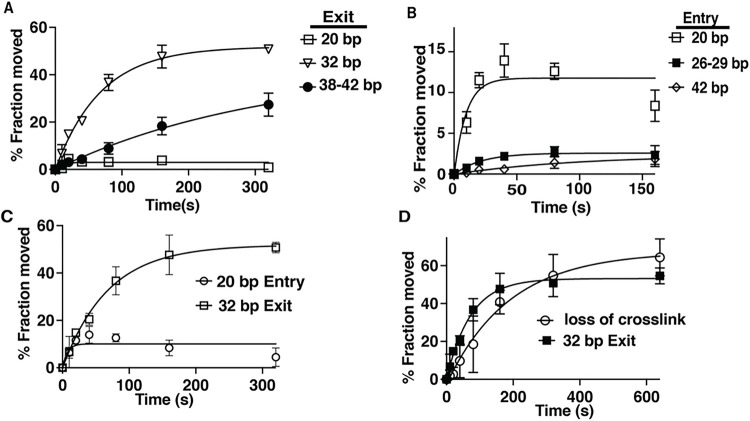
Concerted DNA movement and displacement occur during INO80 remodeling. (A -B) The distance and extent of DNA moved is plotted for the (A) exit and (B) entry sides as derived from phosphorimages like that shown in Supplementary Figure 2A. (C) The plot shows the extent of 32 bp of DNA being moved at the exit side versus the amount of DNA moved 20 bp at the entry side. (D) The rate and extent at which 32 bp of DNA is moved out the exit side compared to that of DNA displacement on the entry side is shown. Displacement correlates to loss of DNA crosslinking at the starting position on the entry side without a gain of new DNA positions.

DNA movement during nucleosome remodeling is tracked using a photoreactive reporter attached to residue 53 of histone H2B that crosslinks and cleaves DNA ^18,22^. DNA initially moves 20 base pairs (bp) on the exit side of nucleosomes then rapidly advances an additional 12 bp for a total movement of 32 bp from the starting point, as evident by the 20 bp step being marginally detected and not increasing over time while DNA moving 32 bp continuously increases over a period of 5 minutes (Fig. 2A and Supplementary Fig. 2). Most DNA is moved 32 bp near steady state conditions and a smaller fraction of nucleosomes have DNA that moved 38–42 bp from the starting position (Fig. 2A). On the entry side of nucleosomes, DNA initially moves 11 bp along with a rapid shift to 20 bp from the starting position that stops accumulating after only 20 s and only a minor fraction of DNA is detected that moves farther (Fig. 2B and Supplementary Fig. 2).

For the first 20 s the amount of DNA that moves 32 bp on the exit side is equivalent to the amount of DNA that moves 20 bp on the entry side; however, after 5 minutes the amount of DNA moved 32 bp on the exit side increase 5-fold with no further increase in DNA movement detected on the entry side (Fig. 2C). These data raise the question as to what is occurring at the entry side while DNA movement proceeds at the exit side and where is the DNA coming from inside nucleosomes to be able to move 32 bp of DNA out the exit side. Over the time interval from 20 to 300 s the increase in DNA moving 32 bp on the exit side parallels the displacement of DNA from H2B on the entry (Fig. 2D). DNA displacement is only observed on the entry side and not on the exit side as previously reported^1^. These findings suggest moving DNA 32 bp on the exit side is coupled to DNA being displaced from the histone octamer on the entry side.

### DNA preference of INO80 is determined by the DNA sequence on the entry side where H2A-H2B dimers bind

**Figure 3:**
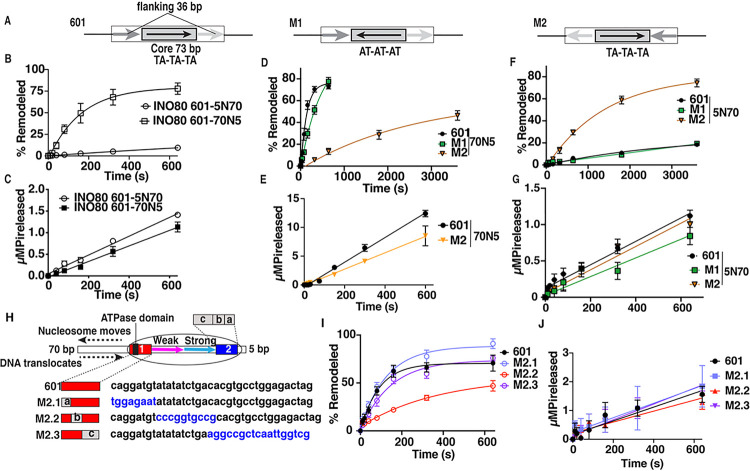
The DNA preference of INO80 is primarily determined by the DNA sequence bound by the H2A-H2B dimer where DNA enters during remodeling. (A) Schematic shows the design for different versions of the 601 nucleosome positioning sequence that have the 72 bp core DNA of 601 flipped (M1) or the 36 bp flanking DNA swapped (M2). (B-C) The rates of (B) nucleosome remodeling as in Supplementary Figure 3A and (C) ATP hydrolysis are plotted for 70N5 and 5N70 nucleosomes with INO80. (D-G) The rate of (D,F) nucleosome remodeling and (E,G) ATP hydrolysis are plotted for M1 and M2 with (D-E) 70N5 nucleosomes and (F-G) 5N70 nucleosomes. Representative gels are in Supplementary Figures 4A-B. (H) The schematic shows the design for dissecting the minimal part of the 36 bp DNA required to inhibit INO80 remodeling. (I-J) The rates of (I) INO80 remodeling and (J) ATP hydrolysis for 601 and three mutated versions of 601 DNA 70N5 nucleosomes are plotted and representative gels are shown in Supplementary Figure 4C. All reactions contained 80 μM ATP, 24 nM INO80 and 8 nM nucleosomes, and were incubated at 30°C. The rate of ATP hydrolysis was determined by thin-layer chromatography. All assays were done in triplicates and error bar denote ±s.d.

We observe a dramatic difference in the rate of nucleosome movement when changing the orientation of INO80 and the direction nucleosomes are moved. We alter the orientation of INO80 on nucleosomes by changing the length of extranucleosomal DNA flanking nucleosomes from 70N5 to 5N70 with the numbers referring to the bp length of extranucleosomal DNA on either side (Fig. 3A). With saturating INO80 and limited ATP, nucleosomes are remodeled > 10 times slower with 5N70 nucleosomes as compared to 70N5 without any significant reduction in the rate of ATP hydrolysis (Figs. 3B–C and Supplementary Fig. 3A). The uncoupling of ATP hydrolysis from nucleosome movement is reminiscent of the effects on INO80 when Arp5 is missing^23–25^ or otherwise unable to bind the acid pocket of the histone octamer^16^.

To delve deeper into this DNA sequence specificity, we switch different sections of the nucleosomal DNA by first reversing the central 72 bp DNA (referred to as M1) that binds to the H3-H4 tetramer and then separately switching the flanking 36 bp of DNA (referred to as M2) that bind to the H2A-H2B dimer (Fig. 3A). There are 3-TA dinucleotides spaced ~ 10 bp apart on the (+) side of the central 72 bp region that contribute to the asymmetric nature of the 601 nucleosome and facilitate in this DNA region being intrinsically curved and energetically favored for binding to the histone octamer surface, while the other half is more rigid ^26^. These nucleosome modifications were then tested in both the 70N5 and 5N70 configurations.

The outcomes of these experiments are quite revealing. Altering the flanking DNA (M2) has a more pronounced effect on INO80 nucleosome mobilization compared to changing the orientation of the core DNA (M1). Depending on the orientation of INO80 switching the flanking 36 bp DNA either stimulated or repressed nucleosome movement with 70N5 M2 nucleosomes being remodeled 16-fold less efficiently and the rate of remodeling of 5N70 M2 nucleosomes increasing at least 5-fold as estimated after remodeling for 30 minutes (Table 1, Figs. 3D and F and Supplementary Fig. 3E-F). These two independent results clearly show the DNA sequence where H2A-H2B binds on the entry side is a primary factor for the DNA sequence specificity of INO80. In contrast reversing the orientation of the central 72 bp had no significant effect on INO80 remodeling (Figs. 3D and F, Supplementary Figs. 3E-F and 4A-B, and Table 1). The difference in the rate of mobilizing nucleosomes using M2 is not caused by changes in the rate of ATP hydrolysis and reflect the same effects seen between 601 containing 70N5 and 5N70 nucleosomes (Fig. 3E and G, Supplementary Figs. 3D and 4A-B, and Table 2). Switching the flanking 36 bp DNA in 70N5 nucleosomes also did not perturb the affinity of INO80 for nucleosome as shown in gel shift assays (Supplementary Fig. 3B).

Incorporation of the histone variant H2A.Z in place of H2A does not cause any differences in INO80’s DNA sequence specificity and shows that DNA sequence specificity does not depend on the type of H2A present (Supplementary Figure S5A-C). Next, we determine if the DNA sequence specificity of INO80 is shared by other ATP-dependent remodelers that sense linker DNA length and space nucleosomes by comparing the properties of ISW2 to INO80. We noticed that while ISW2 did show some DNA sequence-dependent effects, they were generally less pronounced and opposite to what we observed with INO80 and demonstrates the uniqueness of INO80’s DNA specificity (Supplementary Fig. 6).

### The interactions of the ATPase domain at SHL-6 contribute to the DNA sequence-specificity of INO80 chromatin remodeling.

We endeavor to find the minimal DNA required for conferring the DNA sequence specific effects on INO80 remodeling by making hybrids of the 36 bp DNAs that inhibit and promote INO80 remodeling into the entry side of nucleosomes (Fig. 3H). We start with the 36 bp DNA that activates INO80 remodeling and replace short segments of this region with DNA from the inhibitory 36 bp DNA. Replacing the A/T rich region at SHL-6 with the G/C rich part of the inhibitory DNA (M2.2) reduced the rate of nucleosome movement by 4–5-fold for INO80; whereas replacing 8 bp at the entry site (M2.1) or 18 bp at SHL-3/-4 (M2.3) of the 36 bp inhibitory DNA had no negative impact on remodeling (Fig. 3I, supplementary Fig. 4C and Table 1). It is expected that changing the DNA sequence and potentially DNA rigidity where the ATPase domain binds at SHL-6 could affect the propensity of INO80 to pull DNA away from the histone octamer as observed in the cryo-EM structure ^6,7^. The rate of ATP hydrolysis with all three mutant nucleosomes are equivalent to the original 601 nucleosomes and indicates that the reduction of remodeling observed with replacing the 10 bp DNA (M2.2) is uncoupled from ATPase activity, consistent with earlier observations (Fig. 3J and Table 2).

### DNA sequence dependent changes in the interactions of Arp5 and the ATPase domain of Ino80

**Figure 4:**
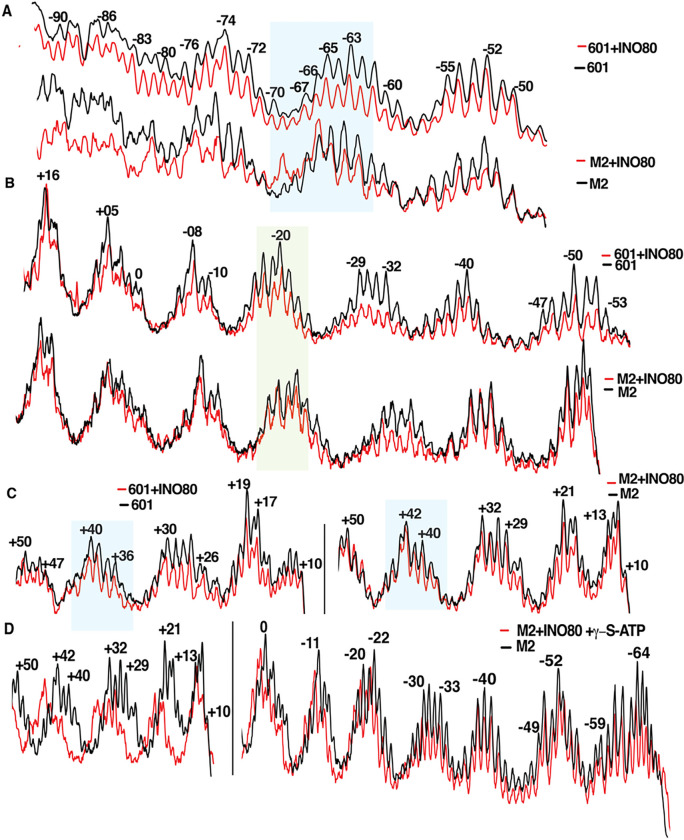
DNA sequence at the H2A-H2B dimer proximal to the entry site alters binding of INO80 to nucleosomal DNA (A-C) The interactions of INO80 with 601 and M2 70N5 nucleosomes is probed by DNA footprinting at the entry sides of (A) lower and (B) upper DNA strands and (C) on the upper strand at the exit side. (D) Initial translocation of INO80 on the upper strand at the exit and entry sides is tracked by DNA footprinting using M2 70N5 nucleosomes with g-S-ATP. The DNA footprint for M2 nucleosomes alone (black) and with INO80 (red) are overlaid for comparison. The blue and green highlights respectively indicate where the ATPase domain of Ino80 and the Arp5 subunit are known to be bound.

To explore why M2 nucleosomes are remodeled less efficiently, we used DNA footprinting and find most of INO80’s interactions with DNA are not affected by changes in DNA sequence. The ATPase domain generally remains bound in the vicinities of SHL-5/-6 and SHL + 2/+3 as seen by DNA protection; however, there are subtle changes in the DNA protection pattern indicating a perturbation of how the ATPase domain is bound at SHL-6. Changes are seen on the lower DNA strand of M2 nucleosomes with DNA lifting off from the histone octamer at nt −69 to −70, evident by increased accessibility (Fig. 4A). There is also a partial loss of the ATPase domain binding seen by a loss of protection with M2 nucleosomes on the lower strand at nt −65 to −67 and on the upper strand at nt −52 to −53 (Supplementary Fig. 7A). Binding of the ATPase domain on the exit side of M2 nucleosomes is also altered as seen by shifts in protection on both DNA strands at SHL + 3 and + 4, while retaining the same protection pattern at SHL + 2 (Fig. 4C and Supplementary Fig. 7C). The DNA binding domain of Arp5 also retains binding to SHL-3, but on both strands at SHL-2 its binding is lost in M2 nucleosomes (Fig. 4B and Supplementary Fig. 7B). There are indications from DNA footprinting that binding of the ATPase domain and the DNA binding domain of Arp 5 are altered when bound to M2 nucleosomes.

Next, we observe by DNA footprinting that DNA translocation occurs in the vicinity of SHL + 2 with M2 nucleosomes similar to 601 nucleosomes when g-S-ATP is added (compare Fig. 4D to Fig. 1F). DNA translocation however proceeded farther on M2 nucleosomes, traversing the dyad axis until reaching the SHL-2 position and may be linked to the loss of Arp5 binding at SHL-2 observed by DNA footprinting. These data point to a defect in INO80 regulating DNA translocation when remodeling M2 nucleosomes.

Additionally, we employ histone photocrosslinking to examine Arp5 interactions near the acid pocket region at residue 89 of H2A and 109 of H2B and with histone H3 at residue 80 and H4 at residue 56 (Supplementary Fig. 8A). Arp5 interactions near the acidic pocket appear not to be changed; however, its interaction with H3, close to the SHL2 position on DNA, is reduced when INO80 is bound to M2 nucleosomes (Supplementary Fig. 8). Furthermore, we find a loss of Ies6 crosslinking at residue 80 of H3 but not at residue 109 of H2B. These differences in Arp5 photocrosslinking were not observed at residues 109 of H2B or 56 of H4, confirming the site specificity of Arp5 loss. DNA footprinting and histone crosslinking indicate two changes in INO80 interactions: altered binding of both the ATPase domain and the Arp5/Ies6 module with the histone octamer and nucleosomal DNA that is tied to DNA sequence changes at the H2A-H2B interface.

### DNA sequence at the H2A-H2B interface profoundly affects DNA movement on the entry side of nucleosomes.

**Figure 5:**
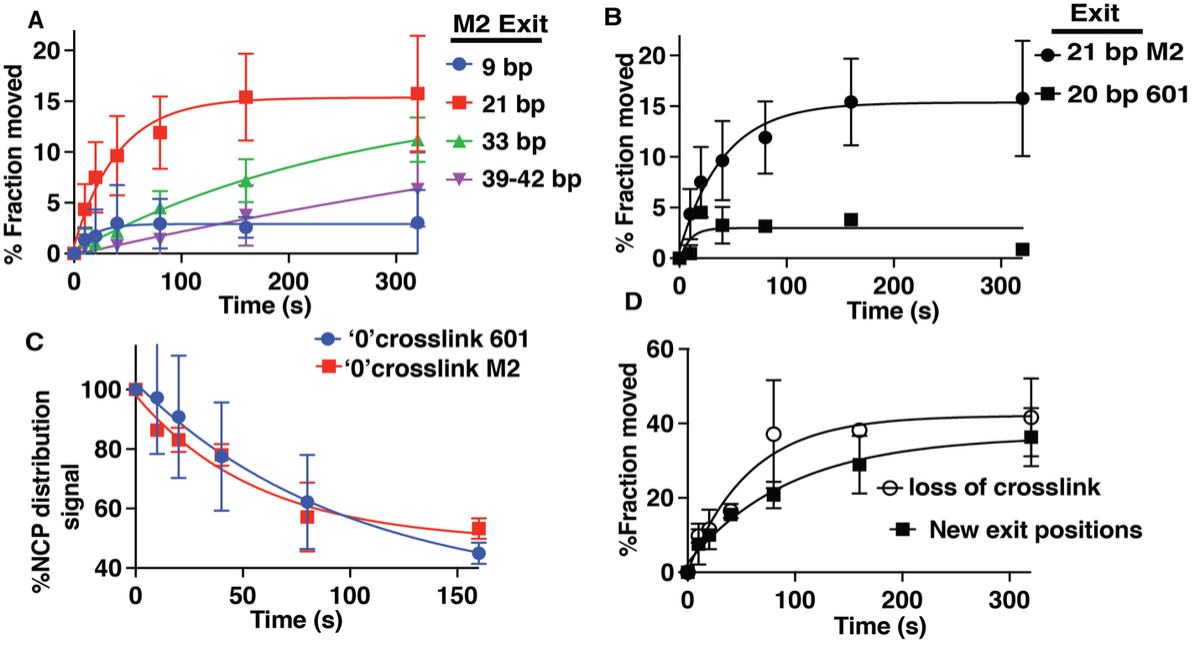
Movement of nucleosomal DNA by INO80 on the entry side is restricted when the DNA sequence is changed at the proximal H2A-H2B interface (A) The extent DNA is moved on the exit side of M2 70N5 nucleosomes is plotted versus reaction time for DNA moved to different distances from the starting position as indicated, similar to Figure 2. (B) The rate at which DNA is moved 20 or 21 bp on the exit side for M2 versus 601 70N5 nucleosome is shown (see also Supplementary Figure 9). (C) The rates at which DNA is displaced from the entry side of nucleosomes are shown for 601 and M2 70N5 nucleosomes by plotting the percent loss of DNA crosslinking/cleavage at the initial starting position versus reaction time. (D) The rate of DNA being displaced at the entry side is compared to the rate of DNA being translocated on the exit side of M2 70N5 nucleosomes by plotting the percent loss of crosslinking/cleavage at the initial starting point on the entry side versus the cumulative of new DNA positions occurring on the exit side versus time. All of these experiments had 3 technical replicates and error bars denote ± s.d.

We map DNA movement during remodeling as described earlier using DNA photocrosslinking and cleavage to discern at which stage in remodeling is DNA sequence important. The first obvious difference when remodeling M2 nucleosomes is that DNA movement on the entry and exit sides of nucleosomes are uncoupled from each other with new DNA positions only detected on the exit side (Fig. 5A and Supplementary Fig. 9A). These findings are consistent with the data described earlier showing that the ATPase domain translocates on DNA at SHL + 2 on the exit side and not at SHL-6. The most significant movement observed on the exit side is a 20 bp step that continues to accumulate up to 160 s, approximately 10 times longer and to an extent 6 times greater than 601 nucleosomes (Fig. 5B). A short step of only 9 bp is also seen, but doesn’t accumulate and seems to rapidly transition to 20 bp. There is a defect in remodeling at the exit side as DNA is unable to move farther than 20 bp in contrast to the primary step size of 32 bp observed with 601 nucleosomes (compare Fig. 5A with Fig. 2A).

The question arises as to where does the 20 bp come from to translocate out the exit side if no DNA movement is observed at the entry side of nucleosomes. We find that DNA displacement occurs on the entry side of M2 nucleosome, equivalent to that observed with 601 nucleosomes, as indicated by reduced crosslinking to DNA not coupled to DNA movement (Fig. 5C). DNA displacement is specific to the entry side and is not observed at the exit side, similar to that observed with 601 nucleosomes (Supplementary Fig. 9B-C). These data suggest DNA displacement at the entry side is not due to DNA translocation at SHL-6 given there are no DNA translocation steps detected, but rather due to translocation at SHL + 2 in contrast to earlier proposed models. Interestingly, the amounts of nucleosomes that have DNA translocating on the exit side equals approximately the same amounts of nucleosomes where DNA is displaced from the entry site, consistent with these two actions being coupled (Fig. 5D). These data show that early steps in INO80 remodeling, including DNA translocation at the exit side and DNA displacement at the entry side, are not adversely modulated by changes in DNA sequence. The step that is regulated however by DNA sequence is likely the subsequent step where more DNA needs to pass from the entry to exit side so that DNA translocation can proceed farther on the exit side (Supplementary Figure. 10).

## Discussion

The model of INO80 engaging and remodeling nucleosomes needs to be substantially revised starting with the ATPase domain’s interaction and translocation on nucleosomal DNA. DNA photocrosslinking and peptide mapping reveals the ATPase domain of Ino80 contacts both SHL-6 and SHL + 2, bridging these two superhelical locations. It becomes evident from DNA footprinting and crosslinking that Ino80 binds simultaneously to both positions as indicated by the extent of the footprint at these positions and the distinct regions of the ATPase domain that are crosslinked. Several approaches are used to decipher at which of these two sites is the cleft region of the ATPase domain bound and therefore translocating on when ATP is added. Both structural modeling of where the ATPase domain is crosslinked to DNA and mapping where DNA translocation occurs when an ATP is analog is added indicates the active cleft region of the ATPase domain is bound and translocates on DNA at SHL + 2 rather than the previously stated SHL-6 position. These findings resolve two previous conundrums as to why the ATPase domains of SWR1 and INO80 complexes engage nucleosomes so differently when they are paralogs and share many of the same subunits and the observation in the original cryo-EM structures of human and *Chaetomium thermophilum* of the ATPase domain engaging either SHL-6 or SHL + 2. Based on our findings, SWR1 and INO80 complexes likely engage nucleosomes more similarly than thought and the observed binding of the ATPase domain at SHL + 2 for INO80 is not an artifact of cryo-EM.

More evidence for the ATPase domain of Ino80 actively translocating on DNA at SHL + 2 comes from the DNA sequence specificity of INO80 providing an effective way to capture early stages in INO80 remodeling. When INO80 nucleosome remodeling is dramatically slowed due to changes in the DNA sequence, we see DNA only be moved to new positions on the exit side of 70N5 M2 nucleosomes. Although no new DNA positions are observed on the entry side of nucleosomes, DNA is displaced where H2A-H2B binds at the same rate and extent as DNA translocation on the exit side. These data suggest DNA displacement near where the ATPase domain binds SHL-6 is likely coupled to DNA translocation at SHL + 2 and not due to DNA translocation at SHL-6 as originally proposed ^1,27^

Next, DNA translocation is arrested after moving 20 bp on the exit side and uncouples the normally fast transition from moving 20 to 32 bp observed with the preferred DNA substrate (70N5 601 nucleosomes). These results indicate that the movement of 32 bp of DNA on the exit side occurs after DNA is displaced from the histone octamer on the entry side and this transition is the stage at which DNA sequence influences the rate of nucleosomes being moved by INO80 (Supplementary Fig. 10).

There are several clues as to how DNA sequence might alter INO80’s binding to nucleosomes and thereby effect its ability to mobilize nucleosomes based on DNA footprinting and site-directed histone crosslinking. Binding of the ATPase domain at SHL-6 is altered by DNA sequence as seen with a loss of INO80 protection at SHL-5 on one strand of DNA and on the other strand at SHL-6 along with DNA lifting off from nt −65 to −67. The ATPase domain still binds to part of DNA at this region and is not completely lost consistent with some type of conformation change. The importance of the ATPase domain binding at SHL-6 for regulating INO80’s DNA sequence specificity is validated by replacement of a 10 bp A/T rich DNA centered at SHL-6 with a G/C rich DNA decreasing the rate of nucleosome movement by a factor of 4–5. These data show the interactions of the ATPase domain at SHL-6 is an important part of the DNA sequence sensing ability of INO80. The ATPase domain of other remodelers including the SWR1 complex are known to have a secondary binding site at SHL6 while also primarily binding to SHL2, as observed here with INO80^10,28–33^. In contrast to these other remodelers, we find that the contacts of the ATPase domain at SHL-6 is crucial for INO80’s DNA sequence specificity and likely vital for INO80’s positioning of + 1 nucleosomes near transcription start sites in vivo, while with these other remodelers only modest effects have been conferred on remodeling^31–33^.

There appears to be more factors involved in the DNA sequence dependence of INO80 because replacement of the 10 bp of DNA at SHL-6 does not show the same extent of inhibition as seen by swapping the 36 bps of DNA bound to the H2A-H2B dimer. It appears likely the Arp5 subunit of INO80 is involved and we observe like the ATPase domain of Ino80 that Arp5 contacts are also DNA sequence dependent. The DNA binding domain of Arp5 binds differently to nucleosomal DNA when the sequence is changed as seen with a loss of protection at SHL-2 on both DNA strands. Second, we observe by site-directed histone crosslinking a loss of Arp5 crosslinking to residue 80 of histone H3 in M2 nucleosomes compared to 601. These changes indicate Arp5 interactions with nucleosomal DNA are perturbed in a DNA-sequence dependent manner rather than through a complete loss of their binding. The involvement of Arp5 is consistent with its previously suggested role as a “gatekeeper” that regulates DNA movement from one side to the other of nucleosomes^7^. The observation with M2 nucleosomes of DNA translocation at SHL + 2 aberrantly moving pass the dyad axis till the SHL-2 position is consistent with the loss of Arp5 contacts at SHL-2 and Arp5 restraining DNA movement between the two sides of nucleosomes.

In summary, our data indicates the DNA sequence specificity of INO80 resides principally at the H2A-H2B region proximal to where linker DNA enters nucleosomes, the ATPase domain of Ino80 and the Arp5 subunit are involved, and the sequence impacts the ability of DNA to pass from the linker proximal to distal sides of the nucleosome. It seems that not only is DNA sequence important, but also the integrity of the DNA strand in this region of the nucleosomes based on earlier studies^1^. We had previously interpreted DNA gaps in the SHL-2 to −6 region that interfere with INO80 mobilizing nucleosomes to be due to blocking DNA translocation^1^. Based on the current data, we realize this is incorrect because INO80 is not translocating on DNA at this region and the gaps instead are likely tied to the strong DNA dependence observed at this same location. Together these data suggest the physical properties of the DNA being displaced from the histone octamer, including flexibility/rigidity, are crucial for regulating the rate of nucleosome mobilization by INO80 which are impacted by DNA sequence or breaks in the DNA strand. Studies from Phillip Korber, Karl-Peter Hopfner and Sebastian Eustermann have shown that INO80 with yeast chromatin reconstituted in vitro preferentially stops mobilizing nucleosomes when encountering DNA that is highly enriched with a less negative propeller twist from SHL-3 to SHL-6, indicative of DNA flexibility at this region being important for the specificity of INO80 to position nucleosomes^14^. These data are consistent with our observations and further highlight the importance of DNA physical properties in this region. More work is needed to better understand how DNA shape effects this important step in INO80 remodeling.

This revised understanding of INO80’s mechanism raises questions about whether hexasomes are the correct substrate for INO80 and if hexasomes remodeled by INO80 would exhibit the same DNA sequence specificity as nucleosomes, given the importance of the DNA bound to the proximal H2A-H2B dimer that is typically absent in the hexasome experiments^34^.

## Materials and Methods

### Immunoaffinity purification of the INO80 and ISW2 complexes

Native wild-type *Saccharomyces cerevisiae* INO80 and ISW2 complexes were purified by immunoaffinity purification with 2 - FLAG (DYKDDDDK) epitopes attached at the C-terminus of the catalytic subunit. Yeast was grown in 15L YPD cultures (1% yeast extract, 2% peptone, 2% dextrose, 40 ppm antifoam A and 0.05% adenine sulfate), until the OD600 reached 5–6. Cells were harvested, washed with ice-cold water followed by ice-cold H-0.3 buffer (300 mM NaCl, 25 mM Na-Hepes pH 7.8, 0.5 mM EGTA, 0.1 mM EDTA, 2 mM MgCl2, 20% glycerol, and 0.02% NP-40) containing protease inhibitors (1 mM PMSF, 1 mM β-mercaptoethanol, 0.5 Na-metabisulphite, 2 μM pepstatin, 0.6 μM leupeptin, 2 mM benzamidine, and 2 μg/ml chymostatin). The cell pellet was collected and passed through a syringe to make yeast spaghetti, which were frozen in liquid nitrogen. The spaghetti was ground into fine powder by cryogenic grinding using (Spex freezer mill 6870). The yeast powder was suspended with ice-cold H-0.3 buffer with protease inhibitors, and nuclear proteins were extracted (S-100 extract) by ultracentrifugation at 100,000 g for 1 h using a Ti-55.2 rotor in an ultra-centrifuge (Thermo Fisher) at 4°C. The supernatant containing the soluble protein fraction from ultracentrifugation was incubated overnight at 4°C with the Anti-FLAG M2 agarose beads (Sigma Aldrich) (10 μl beads per ml S-100 extract) equilibrated with buffer H-0.3 with protease inhibitors. The resin was washed several times with buffer H-0.5 followed by H-0.1 (same composition as H-0.3, but containing 500 mM and 100 mM of NaCl respectively) with protease inhibitors. FLAG-tagged protein complexes were eluted from the resin with 1 mg/mL solution of 3X-FLAG peptide in buffer H-0.1 containing PMSF only (no other protease inhibitor). Complex purity and integrity was determined by analyzing samples on 4–20% gradient SDS–polyacrylamide gels and staining with coomassie brilliant blue R-250 and SYPRO Ruby protein stain.

### 601 DNA constructs preparation

The M1 and M2 601 DNA constructs were synthesized by IDT-DNA. The M1 construct was generated by swapping and taking the reverse complement of the middle 36 bp DNA fragments, whereas the M2 construct was generated by swapping and taking the reverse complement of the outer 36 bp DNA fragments. The 601 DNA sequence mutant M2.1, M2.2, and M2.3 constructs were generated by site directed mutagenesis using the NEB SDM Kit by swapping the nucleotide regions with the opposite M2 corresponding nucleotide regions.

**Table:** 

> 601 TTTCCCAGTCACGACGTTGTAAAACGACGGCCAGTGAATTCGAGCTCGGTACTCGGGTTCAATACATGCACAGGATGTATATATCTGACACGTGCCTGGAGACTAGGGAGTAATCCCCTTGGCGGTTAAAACGCGGGGGACAGCGCGTACGTGCGTTTAAGCGGTGCTAGAGCTTGCTACGACCAATTGAGCGGCCTCGGCACCGGGATTCTCTCGGGGA
>M1 TTTCCCAGTCACGACGTTGTAAAACGACGGCCAGTGAATTCGAGCTCGGTACTCGGGTTCAATACATGCACAGGATGTATATATCTGACACGTGCCTGGAGACTAGTAGCAAGCTCTAGCACCGCTTAAACGCACGTACGCGGTGTCCCCCGCGTTTTAACCGCCAAGGGGATTACTCCCGACCAATTGAGCGGCCTCGGCACCGGGATTCTCCAGGGCG
>M2 TTTCCCAGTCACGACGTTGTAAAACGACGGCCAGTGAATTCGAGCTCGGTACTCGGGTTCAATACATGCATGGAGAATCCCGGTGCCGAGGCCGCTCAATTGGTCGGGAGTAATCCCCTTGGCGGTTAAAACGCGGGGGACAGCGCGTACGTGCGTTTAAGCGGTGCTAGAGCTTGCTACTAGTCTCCAGGCACGTGTCAGATATATACATCCTGGGGCG
>M2.1 TCCCCGAGAGAATCCCGGTGCCGAGGCCGCTCAATTGGTCGTAGCAAGCTCTAGCACCGCTTAAACGCACGTACGCGCTGTCCCCCGCGTTTTAACCGCCAAGGGGATTACTCCCTAGTCTCCAGGCACGTGTCAGATATATATTCTCCATGCATGTATTGAACCCGAGTACCGAGCTCGAATTCACTGGCCGTCGTTTTACAACGTCGTGACTGGGAAA
>M2.2 TTTCCCAGTCACGACGTTGTAAAACGACGGCCAGTGAATTCGAGCTCGGTACTCGGGTTCAATACATGCACAGGATGTCCCGGTGCCGCACGTGCCTGGAGACTAGGGAGTAATCCCCTTGGCGGTTAAAACGCGGGGGACAGCGCGTACGTGCGTTTAAGCGGTGCTAGAGCTTGCTACGACCAATTGAGCGGCCTCGGCACCGGGATTCTCTCGGGGA
>M2.3 TTTCCCAGTCACGACGTTGTAAAACGACGGCCAGTGAATTCGAGCTCGGTACTCGGGTTCAATACATGCACAGGATGTATATATCTGAAGGCCGCTCAATTGGTCGGGAGTAATCCCCTTGGCGGTTAAAACGCGGGGGACAGCGCGTACGTGCGTTTAAGCGGTGCTAGAGCTTGCTACGACCAATTGAGCGGCCTCGGCACCGGGATTCTCTCGGGGA

### Nucleosome reconstitution

Mononucleosomes were reconstituted with 1.7 μg of PCR-generated 70N5 and 5N70 DNA (70 and 5 bp of flanking DNA on either side of 145 bp of 601 nucleosome position sequence DNA) and 100 fmol of 5 ´[^32^P]-labeled DNA at 37°C by rapid salt-dilution with 3–5 μg of recombinant *Xenopus laevis* histone octamers (wild-type or cysteine mutant octamer, see below). The DNA and histone octamer was serially diluted from 2 M to 280 mM NaCl in steps at 37°C. Reconstituted nucleosomes were examined on a native 4% polyacrylamide gel (35.36 acrylamide: 1 bisacrylamide) (PAGE) and the ^32^P signal was captured on phosphorimaging (Typhoon FLA 9500 laser scanner, GE Healthcare Life Sciences).

### Nucleosome remodeling

Nucleosomes 70N5 or 5N70 (8 nM) were bound with saturating amounts of INO80 (24 nM) and ISW2 (18 nM) at 30°C for 30 min. Nucleosome sliding was initiated by adding ATP to a final concentration of 80 μM for INO80 remodeling and 10 μM for ISW2, and incubated at 30°C. Remodeling reactions were stopped at the indicated time points by adding γ-S-ATP and sonicated salmon sperm DNA (stop mix) to final concentrations of 1.5 mM and 300 ng/μl, respectively. Samples were analyzed on 5% native polyacrylamide gels in 0.2X TBE buffer. Data images in figures are representative of 3 experiments. For H2A.Z remodeling amount of nucleosome used was 16–20nM.

### ATPase assay

ATPase assays were performed by initially binding 50nM of nucleosomes and 10nM of INO80 used in each reaction. After binding for 30 min at 30°C, a mixture of ^32^P labeled ATP and unlabeled ATP (Roche) was added to a final concentration of 80μM. Michaelis–Menten kinetics studies were carried out by incubating the reaction for 0, 10, 20, 30, 60, 150, 300, 600, 1200, 3600 seconds at 30°C with the increasing substrate concentrations from 20 uM to 800uM. Reactions were stopped by addition of EDTA and SDS to final concentrations of 100 mM and 2%, respectively. Reactions were spotted onto a polyethyleneimine cellulose plate (J.T. Baker) and resolved with 0.5 M LiCl and 0.5 M formic acid. TLC plate was exposed and visualized by phosphorimaging. Experiments were repeated 3 times.

### Binding Assay

Cy5 labelled DNA template was amplified for 70N5 601 and M2 DNA templates and nucleosomes were reconstituted. An increasing concentration of INO80 complex was incubated with 40nM nucleosome at 30°C for 30 minutes, in 10 mM Na-HEPES (pH7.8), 4 mM MgCl2, 60 mM NaCl, 0.2 mM EGTA, 0.04 mM EDTA and 8% glycerol. Reactions were analyzed by resolving enzyme-bound nucleosomes from free nucleosomes on 5% native polyacrylamide gels in 1X Tris-EDTA buffer. Data images in figures are representative of 3 experiments.

#### High-resolution mapping of changes in H2B53–DNA contacts.

Histone octamers containing cysteine at residue 53 of H2B were conjugated to p-azido phenacyl bromide (APB) immediately after octamer refolding. INO80 was incubated with 70N5 601 and M2 nucleosomes at 30°C for 15min in conditions so that nucleosomes would be completely bound. Nucleosome movement was initiated by adding 80 μM ATP and was stopped with sonicated salmon sperm DNA (300 ng/μl) and excess EDTA at the indicated time points. For site-directed histone-DNA crosslinking, samples were irradiated with UV (3 min at 310 nm, 2.65mW cm - 2). Samples were denatured with 0.1% SDS at 37°C for 20min in 30mM NaCl and 20mM Tris-HCl (pH 8.0). Crosslinked protein-DNA were enriched and separated from un-crosslinked DNA by phenol-chloroform (4:1) extraction. The aqueous phase containing un-crosslinked DNA was discarded. Crosslinked DNA was ethanol precipitated with 1M LiCl in the presence of sheared salmon sperm DNA as carrier. Crosslinked DNA was cleaved with 1M pyrrolidine (Sigma) at 90°C for 15 min. DNA samples were analyzed alongside a sequence ladder made from the same DNA, on denaturing 6.5% polyacrylamide gels containing 8M urea. Gels were visualized by phosphorimaging and quantified using ImageQuant software (Version 5.2). Total lane intensity was normalized to correct for loading bias using Microsoft Excel. Data images in figures are representative of ≥ 3 experiments.

### Hydroxyl radical footprinting

INO80-nucleosome footprinting for nucleosome bound complex, upon addition of ATP analog -γ-S-ATP (incubated for 320s upon addition of final concentration of all at 600 μM) was performed as described ^35^, except that the final concentrations of Fe(II)-ammonium sulfate, H_2_O_2_, Na-ascorbate and EDTA used were 0.3 mM, 0.02%, 6 mM and 0.3 mM, respectively. Cleavage reactions (42 μL) were terminated after 30 s by the addition of 100 μL of termination mix (5 M ammonium acetate, 5 mM thiourea and 10 mM EDTA). DNA was isolated by phenol-chloroform extraction followed by ethanol precipitation at −20 °C. Samples were resolved on a denaturing (8 M Urea) 6.5% polyacrylamide gel with a sequencing ladder created the same DNA template using the SequenaseTM Quick- Denature Plasmid Sequencing Kit from Affymetrix. The gels were dried, phosphorimaged and data analyzed with ImageQuant and Microsoft Excel.

#### Peptide mapping of Ino80 subunit with ArgC protease.

Photoaffinity-labeled INO80 complex (after digestion of DNA and label transfer) was denatured with 0.4% SDS and heating at 90°C for 3 minutes, followed by buffer exchange using Amicon Ultra filters to remove SDS and FLAG peptides. C-terminal FLAG-tagged INO80 was purified by immobilization on ANTI-FLAG^®^ M2 Affinity Gel (Sigma). Protein-bound beads were washed and resuspended in ArgC incubation buffer containing 50mM Tris-HCl (pH 7.8), 5mM CaCl2 and 2mM EDTA. Protein cleavage was initiated by the addition 5mM DTT (final concentration) and varying concentrations of ArgC protease (Promega, sequencing grade) with incubation at 37°C for 2 hours. Reactions were stopped by the addition of 1 mM PMSF and 10mM EDTA. Immobilized C-terminal fragments were separated from the released fragments and washed three times in the same buffer as the digestion. The bead fractions were resuspended in SDS sample buffer, resolved on 4–20% Tris-glycine SDS-polyacrylamide gels, and analyzed by both phosphorimaging, as well as transfer and anti-FLAG immunoblotting (see below). Apparent molecular masses of the Ino80-FLAG fragments were estimated by comparing their migration relative to the [^35^S]-labeled Ino80-FLAG markers of known molecular weights prepared by in vitro coupled transcription and translation using TnT^®^ T7 Quick Coupled Transcription/Translation System (Promega) as described before ^1^. Data images in figures are representative of ≥ 3 experiments.

### Western blots

Western Blots were performed as described before ^1^. Briefly, Protease (ArgC) digested Ino80 fragments were resolved in 4–20% Tris-glycine SDS-polyacrylamide gels and transferred onto PVDF membranes using Bio-Rad Trans-Blot^®^ electrophoretic transfer cell. The membranes were blocked with 5% fat-free milk in TBST (20mM Tris-HCl pH 7.5, 150mM NaCl, 0.1% Tween-20), overnight at 4°C, washed with TBST, and incubated with mouse monoclonal ANTI-FLAG^®^ M2-Peroxidase (HRP) antibody (Sigma Aldrich catalog # A8592) diluted 1:1000 for 1 hour at room temperature. The blots were washed with TBST, and developed with SuperSignal^™^ West Femto Maximum Sensitivity Substrate (Thermo Fisher) visualization using an Image Quant LAS 4000 (GE healthcare Life Sciences). (Supplementary Fig. 1A).

#### Site-specific DNA photoaffinity crosslinking.

Site-specific photo-reactive DNA probes with 601-nucleosome positioning sequence for 70N5 nucleosomes were synthesized by enzymatic incorporation of modified nucleotides into double stranded DNA^36^. dUMP and dCMP analogs coupled with p-azidophenacyl bromide were incorporated along with [α-32P] dGTP/dATP, in tandem, at specific positions. Photo-reactive DNA was reconstituted into nucleosomes and bound with saturating amounts of INO80 at 30°C for 30 min. The extent of enzyme binding was assessed on 4% native polyacrylamide gels. In all experiments > 90% of nucleosomes were bound by the enzyme. After binding, INO80 was crosslinked to DNA by UV irradiation (3 min at 310 nm, 2.65mWcm - 2), and DNA was digested with DNaseI and S1 nuclease for transfer of the radioactive label to the crosslinked protein(s)^36^. Protein subunits were separated on 4–20% SDS polyacrylamide gels and radiolabeled subunit(s) were visualized by phosphorimaging. Data images in figures are representative of ≥ 3 experiments.

### Histone Crosslinking

Recombinant *Xenopus laevis* histones with amino acids replaced by cysteine at specific positions (Supplementary Fig. 8A) were expressed, purified, and reconstituted into octamers with other histones as discussed above. Homogeneous 70N5 601/M2 nucleosomes were reconstituted using the respective 601-nucleosome positioning sequence containing Cy5 label at one end. The PEAS [N-((2-pyridyldithio)ethyl)-4-azidosalicylamide] (Thermo Fisher) -conjugated nucleosomes were iodinated with [^125^I] (Perkin Elmer). Detailed protocol had been discussed and standardized earlier ^16^. Nucleosomes with photoreactive octamers were bound to saturating amounts of INO80 at 30° C and were irradiated directly under UV (3 minutes at 310 nm, 2.65 mW cm-2) for crosslinking. After crosslinking, samples were treated with 100 mM DTT and incubated at 37° C for 30 minutes to transfer [^125^I]-radiolabel to the crosslinked proteins. Samples were resolved on 4–20% SDS-polyacrylamide gels, dried and viewed by phosphor-imaging following 14–16 hours of exposure to identify the radiolabeled subunits. Data images in figures are representative of 3 individual experiments..

## Figures and Tables

**Table 1:** INO80, ISW2 remodeling kinetics table

Enzyme	NCP	5N70 Kmax	70N5 Kmax	5N70 K	70N5 K
INO80	**601**	ND	0.56 avg	ND	7.4 ±1.4 pM/s
	**M1**	ND	0.22	ND	2.1 ±0.96 pM/s
	**M2**	0.057 avg	0.025 avg	0.71 ±0.15 pM/s	0.34 ±0.25 pM/s
	**M2.1**	ND	0.69	ND	7.8 ±1.0 pM/s
	**M2.2**	ND	0.15	ND	2.7 ± 0.80 pM/s
	**M2.3**	ND	0.52	ND	7 ±1.0 pM/s
ISW2	**601**	1.6	3.6	19 ±4.4 pM/s	40 ±7.2 pM/s
	**M1**	6.2	2.1	68 ±7.6 pM/s	22 ± 2.8 pM/s
	**M2**	1.7	1.1	18 ±2.2 pM/s	11 ±2.1 pM/s

**Table 2:** INO80 and ISW2 ATPase kinetics table

Enzyme	NCP	5N70 Slope	70N5 Slope
INO80	**601**	1.8± 0.13 nM/s	21 ± 0.91 nM/s
	**Ml**	1.3 ±0.15 nM/s	2.3 ± 0.18 nM/s
	**M2**	1.7 ±0.13 nM/s	14 ± 1.2 nM/s
	**M2.1**	ND	2.5 ± 1.2 nM/s
	**M2.2**	ND	1.9 ±0.73 nM/s
	**M2.3**	ND	2.7 ±0.75 nM/s
ISW2	**601**	4.7 ±0.28 nM/s	2.7 ±0.27 nM/s
	**M1**	7.4 ±0.61 nM/s	10 ± 1.9 nM/s
	**M2**	32 ± 1.6 nM/s	21 ± 1.8 nM/s
Enzyme	**Vmax(μM/s)**	**Km(μM)**	**Kcat s^− 1^**
INO80	**601**: 0.06	299.6	6.50
	**M2**: 0.08	465.9	8.00
